# Reduced cholesterol levels impair Smoothened activation in Smith–Lemli–Opitz syndrome

**DOI:** 10.1093/hmg/ddv507

**Published:** 2015-12-18

**Authors:** Robert Blassberg, James I. Macrae, James Briscoe, John Jacob

**Affiliations:** 1 The Francis Crick Institute, Mill Hill Laboratory, Mill Hill, London NW7 1AA, UK,; 2 Nuffield Department of Clinical Neurosciences, John Radcliffe Hospital, Level 6, West Wing, Oxford OX3 9DU, UK,; 3 Department of Neurology, Milton Keynes Hospital, Standing Way, Milton Keynes, Buckinghamshire MK6 5LD, UK and; 4 Department of Neurology, John Radcliffe Hospital, Headley Way, Oxford OX3 9DU, UK

## Abstract

Smith–Lemli–Opitz syndrome (SLOS) is a common autosomal-recessive disorder that results from mutations in the gene encoding the cholesterol biosynthetic enzyme 7-dehydrocholesterol reductase (DHCR7). Impaired DHCR7 function is associated with a spectrum of congenital malformations, intellectual impairment, epileptiform activity and autism spectrum disorder. Biochemically, there is a deficit in cholesterol and an accumulation of its metabolic precursor 7-dehydrocholesterol (7DHC) in developing tissues. Morphological abnormalities in SLOS resemble those seen in congenital Sonic Hedgehog (SHH)-deficient conditions, leading to the proposal that the pathogenesis of SLOS is mediated by aberrant SHH signalling. SHH signalling is transduced through the transmembrane protein Smoothened (SMO), which localizes to the primary cilium of a cell on activation and is both positively and negatively regulated by sterol molecules derived from cholesterol biosynthesis. One proposed mechanism of SLOS involves SMO dysregulation by altered sterol levels, but the salient sterol species has not been identified. Here, we clarify the relationship between disrupted cholesterol metabolism and reduced SHH signalling in SLOS by modelling the disorder *in vitro.* Our results indicate that a deficit in cholesterol, as opposed to an accumulation of 7DHC, impairs SMO activation and its localization to the primary cilium.

## Introduction

**Figure 1.**
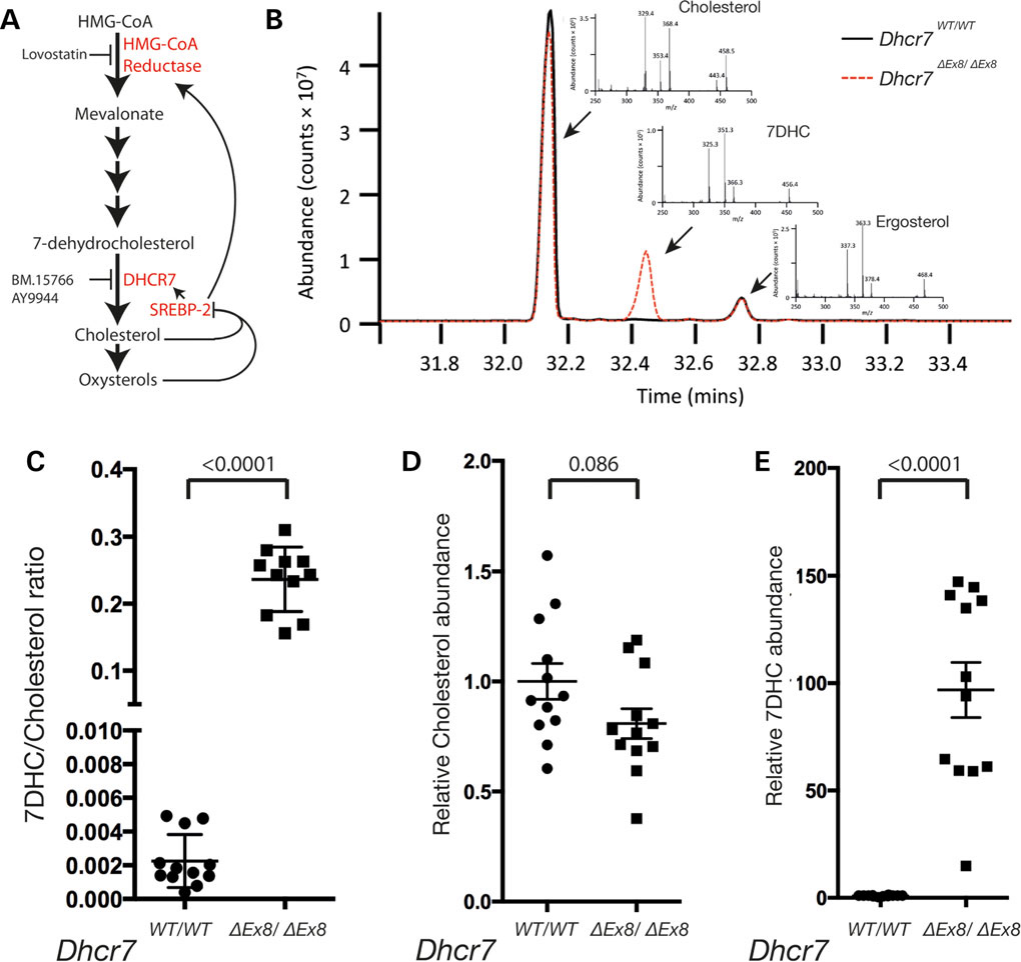
GC-MS analysis of sterol levels. (**A**) Mevalonate is synthesized by HMG-CoA reductase. A series of subsequent metabolic reactions produce the cholesterol precursor and DHCR7 substrate, 7DHC. Cholesterol is a substrate for oxysterols, and both lipids negatively regulate the transcription of metabolic enzymes via SREBP-2. The enzymatic activities of HMG-CoA reductase and DHCR7 are inhibited by the pharmacological compounds Lovastatin, and AY9944 and BM15.766, respectively. (**B**) Example total ion chromatogram illustrates peaks for cholesterol, 7DHC and the internal standard ergosterol. Diagnostic fragment ions of cholesterol, 7DHC and ergosterol used for identification/quantification of each sterol are shown (inset). (**C**) 7DHC/cholesterol ratios for each sample of WT and *Dhcr7^ΔEx8/ΔEx8^* MEFs analysed. (**D**) Relative abundance of cholesterol between samples of WT and *Dhcr7^ΔEx8/ΔEx8^* MEFs. (**E**) Relative abundance of 7DHC between samples of WT and *Dhcr7^ΔEx8/ΔEx8^* MEFs. Bars represent mean ± SEM. *P*-values from unpaired *t*-test.

In humans, cholesterol synthesis in fetal tissues occurs via a series of enzyme driven biochemical steps and is essential for normal development (Fig. 1A). Among the inborn errors of metabolism, disorders of cholesterol metabolism are exceptional because of their strong association with congenital malformations (1). Common to all these conditions is a deficiency in cholesterol and the accumulation of precursor sterols whose identity depends on which enzyme is affected in the biosynthetic pathway. The most common cholesterogenic disorder is Smith–Lemli–Opitz syndrome (SLOS) (2). The birth prevalence of SLOS is estimated to be ∼1/20 000–1/40 000 in Caucasians, making it the third most common autosomal-recessive disorder in these populations (3–5). Affected individuals display growth retardation, developmental delay and a failure to thrive. Congenital abnormalities associated with SLOS affect multiple organs and include cleft palate, syndactyly and polydactyly, neurological defects such as holoprosencephaly (HPE) or microcephaly, and agenesis of the corpus callosum. Dysgenesis of the atrial and ventricular septa of the heart also occurs (6). SLOS patients also exhibit autism spectrum disorder, intellectual disability and electrographic seizures (7–9). Indeed, autistic behaviour may be the only indicator of the disorder in mildly affected individuals (10).

SLOS is caused by recessive mutations in the terminal cholesterol biosynthetic enzyme, 7-dehydrocholesterol reductase (DHCR7) (4,11,12). These perturb its ability to catalyse the conversion of 7-dehydrocholesterol (7DHC) to cholesterol. In Caucasian populations, 60% of SLOS-causing mutations are accounted for by six alleles of *Dhcr7* (13). Of these, the c.964-1G>C splice-acceptor mutation is the most common and accounts for∼30% of mutant alleles reported in SLOS (14–17). The c.964-1G>C mutation produces a frameshift resulting in premature termination of the protein-coding sequence and a functionally null allele (18). Similarly, the W151X allele harbours a non-sense mutation resulting in a truncated non-functional protein (19). Other common alleles associated with SLOS (T93M, R404C, V326L and R352W) result in missense mutations that diminish the enzymatic activity of DHCR7 (14). Carrier frequencies of *Dhcr7* mutations associated with the disorder have been calculated to be in the range of 1–2%, predicting a prevalence significantly higher than observed clinically (20). This discrepancy may be explained by the broad range in the severity of the abnormalities in affected individuals, with the least severely affected remaining unidentified and the most severe resulting in prenatal demise.

Under normal physiological conditions sterol sensing proteins localized in the membrane of the endoplasmic reticulum regulate cellular cholesterol homeostasis through a feedback mechanism involving transcriptional regulation of cholesterol biosynthetic enzymes (21), including 3-hydroxy-3-methylglutaryl-CoA (HMG-CoA) reductase (HMGCR*)* and DHCR7 itself (22,23) (Fig. 1A). Through this mechanism the level of activity of enzymes in the biosynthetic pathway are tuned to ensure that rates of the synthesis of precursors are balanced against the requirement for their products. SLOS-causing mutations not only reduce the level of cholesterol, but also result in elevated levels of its precursor 7DHC (24), which continues to be synthesized in response to reduced cholesterol levels. In healthy individuals 7DHC levels are almost undetectable and an increased ratio of 7DHC/cholesterol is characteristic of SLOS (25).

The morphological features of SLOS overlap those observed as a result of mutations in components of the Sonic Hedgehog (SHH) signalling pathway, suggesting a functional connection (26). SHH signalling is involved in the patterning of many tissues during embryonic development including the skeletal, central nervous and cardiovascular systems, where it determines the fate of progenitor cells in a concentration-dependent manner (27–29). As in SLOS, defective SHH signalling is associated with HPE and dysgenesis of the corpus callosum (30,31), cleft palate (32) and digit (32) and heart malformations (33). SHH signalling is initiated by the binding of SHH to the receptor Patched (PTC), which relieves tonic repression of the transmembrane protein Smoothened (SMO) (34). In mammalian cells SHH pathway signal transduction is associated with a microtubule-associated sensory organelle known as the primary cilium that protrudes from the plasma membrane (35). SHH binding to PTC results in its depletion from the primary cilium (36), translocation of SMO to the ciliary membrane (36–38) and the subsequent accumulation of active GLI proteins that translocate to the nucleus and induce the expression of target genes including *Gli1* and *Ptc* (39).

The mechanism by which PTC represses SMO activation and translocation into the primary cilium in the absence of SHH remains obscure. However, homology between PTC and the Niemann–Pick cholesterol transporter hint at the involvement of sterols (40,41). The plant-derived sterol cyclopamine directly inhibits SMO activity, further suggesting a role for sterols in the regulation of SHH signalling (42,43). It is also becoming clear that specific oxysterols produced by the cholesterol biosynthetic pathway also interact directly with SMO and act as potent activators of SHH signalling (44–46). Activating oxysterols and synthetic inhibitory oxysterol analogues interact with an oxysterol-binding motif located extracellularly at the N-terminal of SMO (47–49), whereas cyclopamine, a naturally occurring SMO inhibitor, and other positively acting sterol compounds such as the synthetic Smoothened agonist (SAG) bind the membrane-spanning heptahelical bundle (50). Thus SMO exhibits at least two distinct, functionally important sterol-interacting interfaces, both of which can either positively or negatively regulate its activity. In addition to the direct interactions between active sterol molecules and SMO, cholesterol levels have been shown to play a permissive role in SMO activation (48).

Despite these recent advances the relationship between cholesterol and SHH signalling remains enigmatic. SLOS is associated with a deficit of cholesterol and the accumulation of 7DHC (24), and altered levels of either metabolite have been proposed to be pathogenic (51–54). A fundamental question that has not been resolved is whether accumulation of 7DHC or lack of cholesterol or its derivatives is responsible for the deficit in SHH signalling in SLOS. Whereas cholesterol and its oxysterol derivatives have been shown to positively regulate SHH signal transduction (44–46,48), an earlier study has demonstrated that 7DHC can inhibit SHH signalling by acting as a precursor for Vitamin D, which has been proposed to mediate the inhibitory effect of PTC on SMO activation (55). Additionally, oxidized derivatives of 7DHC have been shown to be toxic to developing embryos (56,57). Thus, elevated 7DHC levels may have both non-specific toxic effects on embryo development in addition to effects that are specific to SHH signal transduction. Altered expression of the terminal enzyme DHCR7 has also been implicated in the developmental defects associated with SLOS. Inhibition of SHH signalling at or downstream of SMO by DHCR7 independently of its enzymatic function has been reported, and this activity has been localized to its N-terminal region (58,59). The lack of understanding of how the cholesterol and SHH signalling pathways intersect has also hampered the design of appropriate treatment strategies for SLOS. Cholesterol supplementation and statin treatment have both been investigated as treatments that might mitigate the deleterious effects of *Dhcr7* mutation in SLOS patients (60–65). Raising cellular cholesterol levels through dietary supplementation would be predicted to reduce 7DHC levels via transcriptional regulation of biosynthetic enzymes, whereas statin treatment should reduce the synthesis of both metabolites. However, effects reported from clinical studies are variable (60,63–66). Cholesterol supplementation should also reduce the level of DHCR7 expression whereas statin treatment would be expected to increase it, through feedback regulation. For these reasons, it is important to clarify which of the potential molecular interactions between SHH signalling and components of the cholesterol biosynthetic pathway are relevant in SLOS.

One approach to gaining deeper insight into the pathophysiology of SLOS has relied heavily on a mammalian model of SLOS in which rat embryos *in utero* are exposed to competitive inhibitors of DHCR7 enzymatic activity, in particular AY9944 (67), or the structurally unrelated BM15.766 (68). Rat embryos exposed to these compounds exhibit an increased 7DHC/cholesterol ratio as in SLOS, and associated pituitary agenesis and HPE (69–72). AY9944 has also been shown to inhibit transcriptional responses to SHH signalling during the formation of neural tissue (6,42,73). However, as AY9944 also disrupts the trafficking of cholesterol between the various cellular membranes (74–76) and would therefore be predicted to interfere broadly with signal transduction, it is unclear whether inhibition of SHH signalling occurs through its effect on cholesterol synthesis.

To gain further insight into the molecular basis of perturbed SHH signalling in SLOS, we modelled the disorder *in vitro* using primary and immortalized fibroblast cells widely demonstrated to recapitulate *in vivo* Shh signal transduction (36,37,45,47,48,77,78). We studied SHH signalling in mouse embryonic fibroblasts (MEFs) in which the eighth exon of the *Dhcr7* gene has been deleted (*Dhcr7^ΔEx8^*), mimicking the effect of the most prevalent c.964-1G>C SLOS-causing splice-acceptor mutation (79). We also established the utility of BM15.766 as a more reliable pharmacological tool than the widely used AY9944 to probe the molecular basis of SLOS, and used it to complement our investigation of *Dhcr7* mutant MEFs. SHH signalling is reduced following genetic and chemical disruption of DHCR7 activity due to reduced SMO activation. We show that this is due to a deficit of cholesterol unrelated to the biosynthesis of SMO-activating oxysterols, or accumulation of 7DHC. Furthermore, our data suggest that treatment to lower 7DHC levels with statins is unlikely to correct the developmental defects associated with reduced SHH signalling in SLOS.

## Results

### *Dhcr7* mutant MEFs exhibit an aberrant sterol profile as observed in SLOS patients

To investigate whether the developmental defects characteristic of SLOS patients are associated with an aberrant response to SHH, we derived MEFs from a transgenic mouse model of SLOS in which the *Dhcr7* gene had been engineered to mimic the prevalent c.964-1G>C allele that codes for a C-terminal truncated protein devoid of enzymatic activity (79). We developed a gas chromatography-mass spectrometry (GC-MS) protocol to quantify the absolute levels of cholesterol and 7DHC in serum-starved fibroblast samples (Fig. 1B) and demonstrated a dramatic increase in the 7DHC/cholesterol ratio in homozygous transgenic *Dhcr7* MEFs (henceforth denoted *Dhcr7^ΔEx8/ΔEx8^*), as seen in SLOS patients (Fig. 1C). Our analysis indicated a mean 19% reduction in cholesterol levels in *Dhcr7^ΔEx8/ΔEx8^* MEFs when compared with controls (Fig. 1D), whereas 7DHC levels showed a mean 970% elevation (Fig. 1E).

### SHH signalling is perturbed at the level of SMO activation in *Dhcr7^ΔEx8/ΔEx8^*MEFs

**Figure 2.**
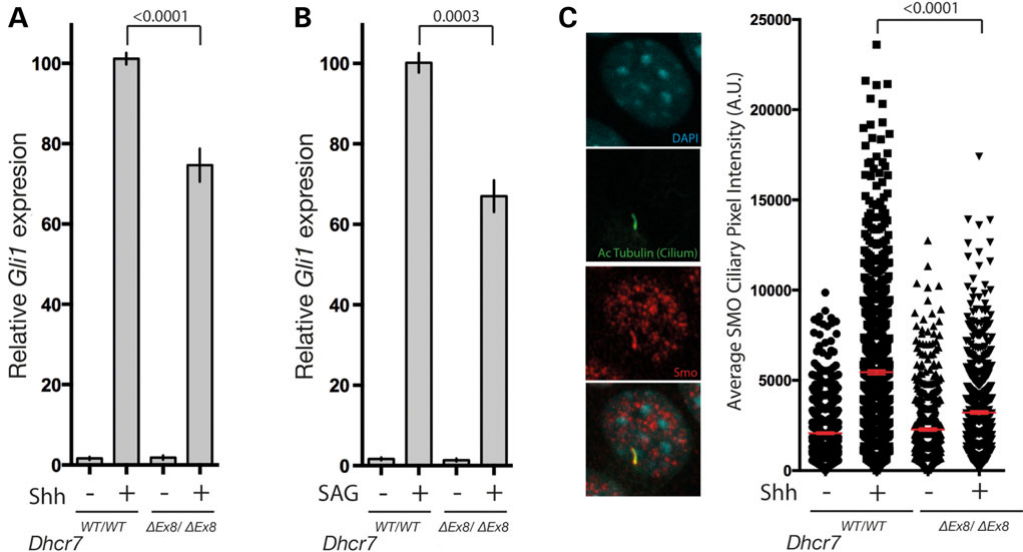
SMO activation is impaired in *Dhcr7^ΔEx8/ΔEx8^* MEFs. (**A**) Gli1 transcriptional response of WT and *Dhcr7^ΔEx8/ΔEx8^* MEFs stimulated with SHH (*n* = 24) or (**B**) SAG (*n* = 8), measured by qPCR. Mean ± SEM. *P*-values from paired *t*-test. (**C**) Immunofluorescence staining of SMO accumulation in primary cilia of SHH-treated WT and *Dhcr7^ΔEx8/ΔEx8^* MEFs. The mean pixel value of individual SMO immunostained cilia was plotted. Bars represent mean ± SEM of mean pixel values. *P*-values from the Kolmogorov–Smirnov test.

Induction of the SHH target gene *Gli1* served as a readout for pathway activity and when compared with MEFs derived from wild-type (WT) littermates, homozygous transgenic *Dhcr7^ΔEx8/ΔEx8^*MEFs exhibited a mean reduction of 26% in response to exogenously supplied SHH (Fig. 2A). The SHH receptor PTC has been proposed to modulate the activity of the pathway signal transducer SMO through the activity of sterol molecules (55,80). To determine whether the defect in the response to SHH observed in *Dhcr7^ΔEx8/ΔEx8^* MEFs is dependent on the activity of PTC we directly activated SMO using the small molecule agonist SAG (77). As SAG stimulated *Dhcr7^ΔEx8/ΔEx8^* MEFs also exhibited a reduced SHH pathway response (Fig. 2B) we propose that the defect in the SHH signalling pathway associated with SLOS is located at or downstream of SMO.

Activation of SMO is associated with its recruitment to a specialized plasma-membrane sub-domain known as the primary cilium within which signal transduction events in the SHH signalling pathway are known to take place (35,81). Consistent with a defect in SMO activation, the reduced SHH pathway response of *Dhcr7^ΔEx8/ΔEx8^* MEFs was associated with a marked reduction in SMO cilia localization in response to SHH (Fig. 2C).

### The small molecule BM15.766 recapitulates the *Dhcr7^ΔEx8/ΔEx8^* mutation

**Figure 3.**
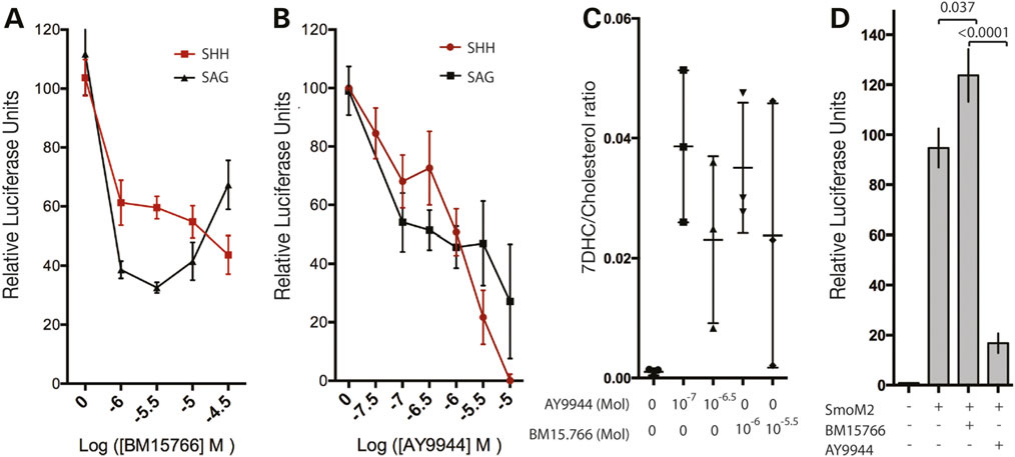
The DHCR7 inhibitor BM15.766 recapitulates the c.964-1G>C mutation. (**A**) The DHCR7 inhibitor BM15.766 partially inhibits activation of a Gli luciferase reporter in Shh-Light2 cells by SHH or direct activation of SMO by SAG across a broad range of concentrations. Mean ± SD (*n* = 3). (**B**) At high concentrations the DHCR7 inhibitor AY9944 completely inhibits activation of a GLI-luciferase reporter by SHH. Mean ± SD (*n* = 6). (**C**) 7DHC/cholesterol ratios derived from GC-MS analysis illustrate that 10^−7^ M AY9944 and 10^−6^ M BM15.766 maximally inhibit DHCR7 enzymatic activity. Mean ± SD. (**D**) Activation of the GLI-luciferase reporter by constitutively active SmoM2 in transfected NIH3T3 fibroblasts is insensitive to 10^−5^ M BM15.766 and inhibited by 10^−5^ M AY9944. Mean ± SEM (*n* = 12). *P*-values from unpaired *t*-test.

We sought a small molecule inhibitor of DHCR7 enzymatic activity to complement our investigation with *Dhcr7^ΔEx8/ΔEx8^* MEFs. The compound AY9944 has been widely used in previous studies modelling SLOS (42,43,70,72,73); however, concerns have been raised about off-target affects, including disruption of the intracellular transport of cholesterol (74–76). For this reason, we reevaluated the appropriateness of AY9944 in modelling SLOS and compared it with a less well-studied small molecule inhibitor of DHCR7 denoted BM15.766 (69,71). For these assays, we employed the Shh-LIGHT2 fibroblast cell line that reports SHH pathway activity via the expression of luciferase controlled by a GLI responsive promoter (78). BM15.766-treated Shh-LIGHT2 cells exhibited a comparable reduction to *Dhcr7^ΔEx8/ΔEx8^* MEFs in their response to SHH and SAG (Fig. 3A) (compare Fig. 2A and B) across a 30-fold range of concentrations. AY9944 reduced SHH pathway activity to a comparable extent as the *Dhcr7^ΔEx8^* mutation when present at 100 nM, however, at 10 μM AY9944 almost completely inhibited signalling following SHH stimulation (Fig. 3B). High concentrations of AY9944 also inhibited the response to SAG (Fig. 3B) more severely than was observed in *Dhcr7^ΔEx8/ΔEx8^* MEFs (compare Fig. 2A and B). As these data indicated a potential effect of AY9944 on SHH signalling that is independent of DHCR7 inhibition, we assessed DHCR7 activity in the presence of the minimal doses of AY9944 and BM15.766 required to mimic the SHH signalling defect observed in *Dhcr7^ΔEx8/ΔEx8^* MEFs. Both 100 nm AY9944 and 1 μm BM15.766 maximally inhibited DHCR7, as shown by an elevated 7DHC/cholesterol ratio that did not increase further at higher concentrations of either inhibitor (Fig. 3C). Taken together, these data indicate that low doses of AY9944 recapitulate the effect of the *Dhcr7^ΔEx8^* mutation, whereas high doses further inhibit SHH signalling independently of its effect on DHCR7 activity.

As experiments in *Dhcr7^ΔEx8/ΔEx8^* MEFs indicated that the defect in SHH signalling operates at the level of SMO activation, we further evaluated how faithfully the DHCR7 pharmacological inhibitors model the effect of the *Dhcr7^ΔEx8^* mutation in relation to a constitutively active SMO receptor. SHH signalling was unaffected by 10 μm BM15.766 in fibroblasts transfected with the constitutively active SmoM2 construct consistent with observations in *Dhcr7^ΔEx8/ΔEx8^* MEFs; however, 10 μm AY9944 strongly inhibited SHH signalling (Fig. 3D). Therefore, pharmacological inhibition of the SHH pathway by BM15.766 much more closely mimics the effect of *Dhcr7* loss of function across a wider range of concentrations than does AY9944. These experiments indicate that BM15.766 is an appropriate reagent to complement studies with *Dhcr7^ΔEx8/ΔEx8^* MEFs and highlight that AY9944 can elicit DHCR7-independent inhibition of SHH signalling at, or downstream of SMO. In light of these observations, we opted to use BM15.766 in further investigations.

### The cholesterol biosynthetic activity of DHCR7 is required for SMO activation

**Figure 4.**
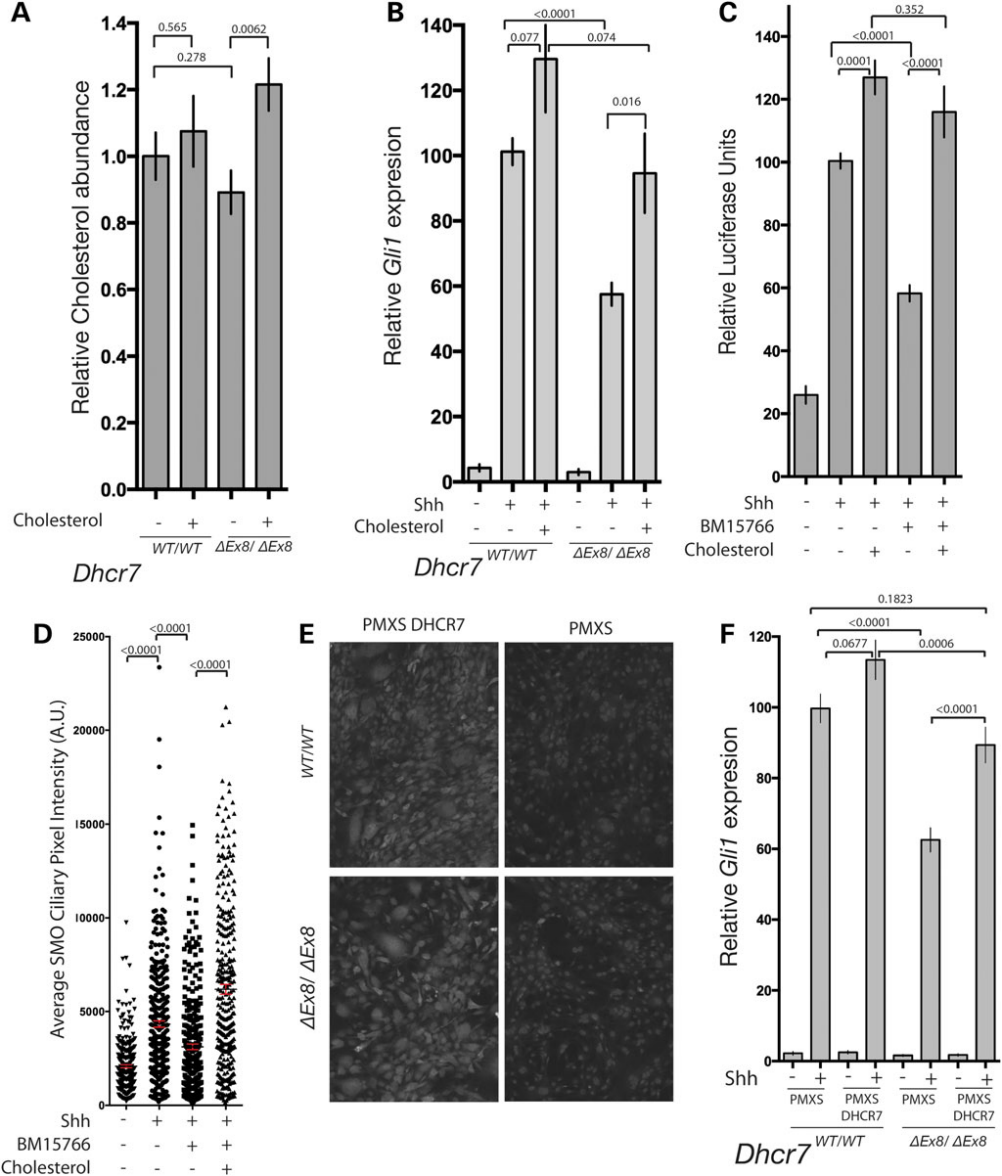
DHCR7 cholesterol biosynthetic activity is required for normal SHH signalling. (**A**) Addition of exogenous cholesterol raised cellular cholesterol levels of *Dhcr7^ΔEx8/ΔEx8^* MEFs as determined by GC-MS. (**B**) *Gli1* transcriptional response of SHH stimulated WT and *Dhcr7^ΔEx8/ΔEx8^* MEFs cultured with or without additional cholesterol. Mean ± SEM. *P*-values from paired *t*-tests (*n* = 10). (**C**) Inhibition of GLI-reporter activation by BM15.766 in Shh-LIGHT2 cells cultured with or without additional cholesterol. Mean ± SEM. *P*-values from the Mann–Whitney test (*n* = 20). (**D**) The mean pixel value of individual SMO immunostained cilia was reduced in SHH-stimulated NIH3T3 fibroblasts cultured with BM15.766. The effect was reversed by cholesterol. Mean ± SEM. *P*-values from the Kolmogorov–Smirnov test. (**E**) Immunofluorescence staining of human DHCR7 overexpressed in MEFs by retroviral transduction compared with untransfected control MEFs. (**F**) DHCR7 overexpression rescued the reduced *Gli1* transcriptional response of SHH stimulated *Dhcr7^ΔEx8/ΔEx8^* MEFs. Mean ± SEM (*n* = 16). *P*-values from paired *t*-tests.

The loss of DHCR7 enzymatic activity in *Dhcr7^ΔEx8/ΔEx8^* mice and SLOS patients has been shown to result in reduced levels of cellular cholesterol (53,79), prompting us to investigate whether reduced cholesterol levels underlie the defective SMO activation we observed. Culturing *Dhcr7^ΔEx8/ΔEx8^* MEFs in the presence of exogenous cholesterol increased cellular cholesterol levels (Fig. 4A) and restored their response to SHH to the level of WT MEFs (Fig. 4B). Cholesterol supplementation also restored the GLI response of BM15.766-treated Shh-LIGHT2 cells stimulated with SHH (Fig. 4C) and rescued the reduced SHH-induced SMO cilia localization (Fig. 4D). Thus, the reduced SMO activation that occurs in the absence of DHCR7 activity is a consequence of reduced cholesterol levels.

*Dhcr7* expression is down-regulated by cholesterol (mean of control ± SD = 51% ± 7.3; SEM = 0.02) and up-regulated by pharmacological inhibition with BM15.766 (mean of control ± SD = 226% ± 28.2; SEM = 0.16) or AY9944 (mean of control ± SD = 225% ± 83.7; SEM = 0.34). As transcriptional up-regulation of DHCR7 due to reduced cholesterol levels has been proposed to underlie the SHH pathway defects in SLOS (58,59), we considered whether rescue of SHH signalling by cholesterol might act via this mechanism. We determined whether the *ΔEx8* mutation in *Dhcr7* led to its transcriptional up-regulation, however contrary to predictions we found a marked reduction in *Dhcr7* transcript levels (mean of control ± SD = 26% ± 5.13; SEM = 0.02), possibly due to non-sense mediated decay of the mutant transcript (82). As *Dhcr7* levels are reduced in *Dhcr7^ΔEx8/ΔEx8^* MEFs, inhibition of signalling by elevated DHCR7 levels is unlikely to underlie the reduced SHH response. To further investigate the relationship between the regulation of DHCR7 protein levels and SHH pathway activity, human DHCR7 protein was constitutively expressed in WT and *Dhcr7^ΔEx8/ΔEx8^* MEFs by viral transduction (Fig. 4E). Whereas overexpression of DHCR7 did not diminish the SHH responsiveness of WT MEFs, it was able to restore signalling in *Dhcr7^ΔEx8/ΔEx8^* MEFs to the level of WT controls (Fig. 4F). We, therefore, conclude that reduced levels of intracellular cholesterol rather than deregulated DHCR7 protein expression perturbs SHH signalling in SLOS.

### Reduced cholesterol levels underlie impaired SMO activation

**Figure 5.**
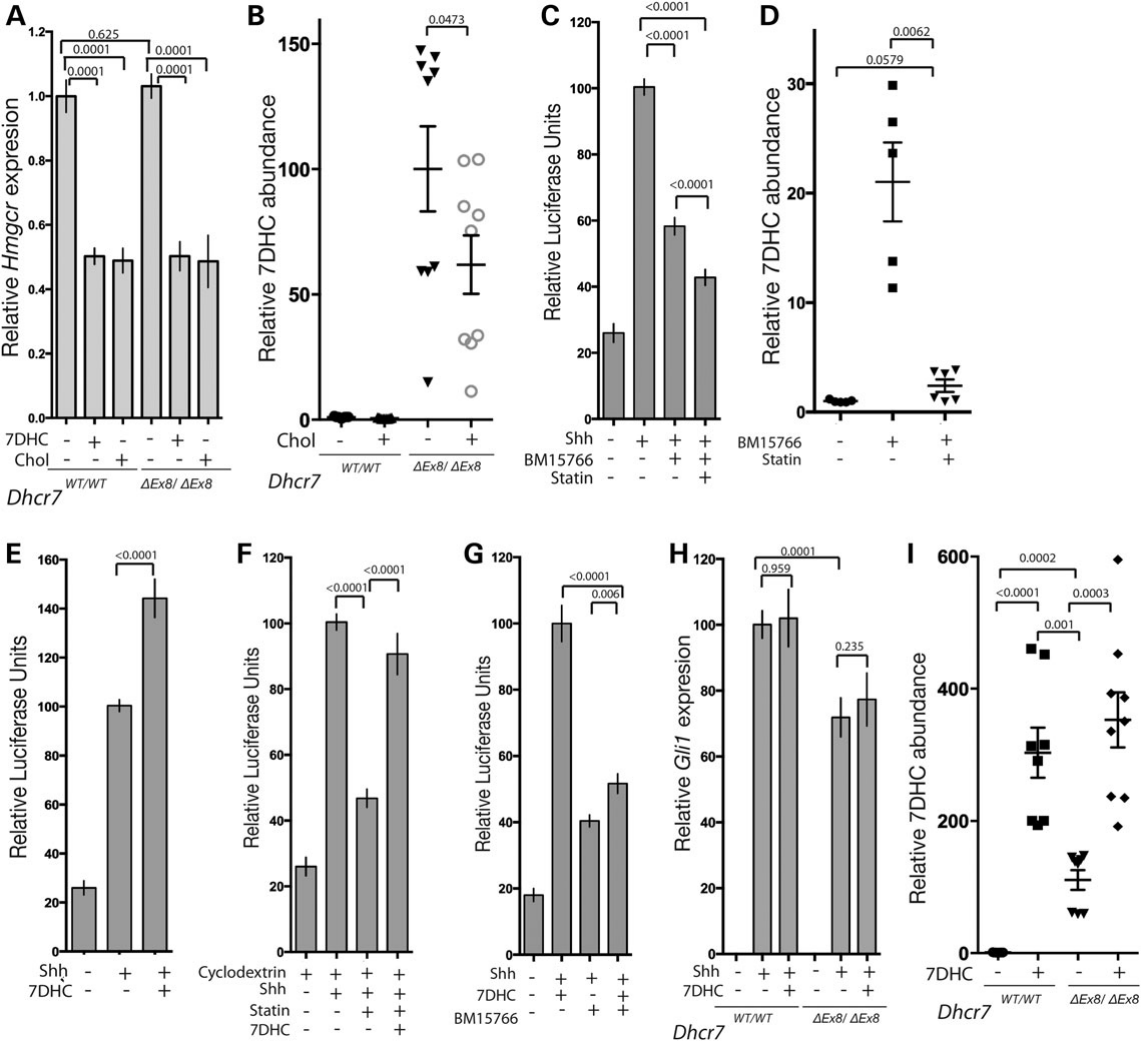
7DHC accumulation does not inhibit SHH signalling. (**A**) *Hmgcr* expression measured by qPCR is equivalent in WT and *Dhcr7^ΔEx8/ΔEx8^* MEFs, and reduced by 7DHC or cholesterol (*n* = 5). (**B**) Exogenous cholesterol reduces the elevated 7DHC levels in *Dhcr7^ΔEx8/ΔEx8^* MEFs. Abundances determined by GC-MS. *P*-values are from paired *t*-tests. (**C**) Inhibition of SHH-induced GLI-reporter activation by BM15.766 in Shh-LIGHT2 cells is not reversed by Lovastatin (*n* = 20). (**D**) Lovastatin blocks 7DHC accumulation in 3T3 cells treated with BM15.766. (**E**) 7DHC did not inhibit SHH-induced GLI-reporter activation (*n* = 20). (**F**) 7DHC rescued reduced GLI-reporter activation by SHH in the presence of 5 uM Lovastatin and 0.1% 2-hydroxypropyl-β-cyclodextrin (*n* = 16). (**G**) 7DHC did not potentiate the inhibitory effect of BM15.766 on SHH-induced GLI-reporter activation (*n* = 20). (**H**) 7DHC had no effect on the *Gli1* transcriptional response of SHH stimulated *Dhcr7^ΔEx8/ΔEx8^* or WT MEFs. Paired *t*-test (*n* = 9). (**I**) Exogenous 7DHC accumulates in WT and *Dhcr7^ΔEx8/ΔEx8^* MEFs. *P*-values are from paired *t*-tests. All panels illustrate mean ± SEM. *P*-values are from Mann–Whitney tests unless stated otherwise.

Reduced levels of cholesterol that result from loss of DHCR7 enzymatic activity are predicted to up-regulate HMGCR through feedback regulation. This would exacerbate the accumulation of the cholesterol precursor, 7DHC that has been proposed to underlie the SHH signalling defects associated with SLOS (55). However, we observed no change in the level of HMGCR expression in *Dhcr7^ΔEx8/ΔEx8^* MEFs (Fig. 5A), consistent with the observation that HMGCR activity is unaltered in SLOS patients (83). As addition of 7DHC down-regulates the expression of HMGCR comparably to cholesterol in both WT and *Dhcr7^ΔEx8/ΔEx8^* MEFs (Fig. 5A), we conclude that in SLOS, elevated 7DHC levels offset the effect of reduced cholesterol levels on HMGCR expression. Nonetheless, 7DHC continues to be synthesized as evidenced by its accumulation in *Dhcr7^ΔEx8/ΔEx8^* MEFs (Fig. 1E) and *Dhcr7^ΔEx8/ΔEx8^* mice (14). As addition of cholesterol down-regulates HMGCR expression, this raises the possibility that the rescue of SHH signalling that we observe in *Dhcr7^ΔEx8/ΔEx8^* MEFs supplemented with cholesterol might be an indirect consequence of reducing the rate of synthesis of 7DHC. In line with this prediction, GC-MS analysis demonstrated a moderate reduction of mean 7DHC levels in mutant cells cultured in the presence of cholesterol (Fig. 5B).

We tested the possibility that cholesterol might indirectly regulate SMO activation by feedback inhibition of the synthesis of 7DHC, by blocking 7DHC synthesis with the HMGCR inhibitor Lovastatin. Lovastatin treatment did not have a positive effect on SHH signalling when DHCR7 activity was inhibited with BM15.766 (Fig. 5C), despite dramatically reducing the accumulation of 7DHC (Fig. 5D). This indicates that the impaired SMO activation resulting from loss of DHCR7 activity is due to reduced levels of cholesterol rather than an accumulation of its precursor, 7DHC. Consistent with this interpretation, we did not observe an inhibitory effect on SHH signalling of culturing cells in the presence of 7DHC. Instead, similar to the addition of cholesterol (Fig. 4C) we observed a moderate potentiation of SHH signalling in Shh-Light2 cells cultured in the presence of 7DHC (Fig. 5E). This could either be due to the loss of the inhibitory activity of 7DHC in our assay following its rapid metabolism to cholesterol by DHCR7, or its genuine lack of inhibitory activity. To distinguish between these possibilities, we performed a series of control experiments. We first demonstrated the activity of 7DHC by its ability to rescue the previously reported reduced SHH response in WT cells cultured in the presence of cyclodextrin and Lovastatin (Fig. 5F) (84). In contrast to cholesterol (Fig. 4C), 7DHC was unable to rescue the SHH response of BM15.766-treated Shh-Light2 cells (Fig. 5G) demonstrating that in the absence of DHCR7 enzymatic activity 7DHC is not converted to cholesterol. In these conditions, we failed to observe further inhibition of SHH signalling when BM15.766-treated cells were exposed to additional 7DHC compared with BM15.766 treatment alone (Fig. 5G). We performed a comparable experiment in WT and *Dhcr7^ΔEx8/ΔEx8^* MEFs and again observed no inhibitory effect of 7DHC in either cell type (Fig. 5H) despite elevation of cellular 7DHC to levels above those observed in untreated *Dhcr7^ΔEx8/ΔEx8^* MEFs (Fig. 5I). As neither reducing 7DHC levels with statin nor elevating them by adding 7DHC under conditions in which they cannot be metabolized to cholesterol showed any detectable effect on SMO activation, we propose that elevated 7DHC levels do not underlie the reduced SHH signalling that we observe in our *in vitro* models of SLOS.

### Cholesterol is required for SMO activation independently of oxysterol production

**Figure 6.**
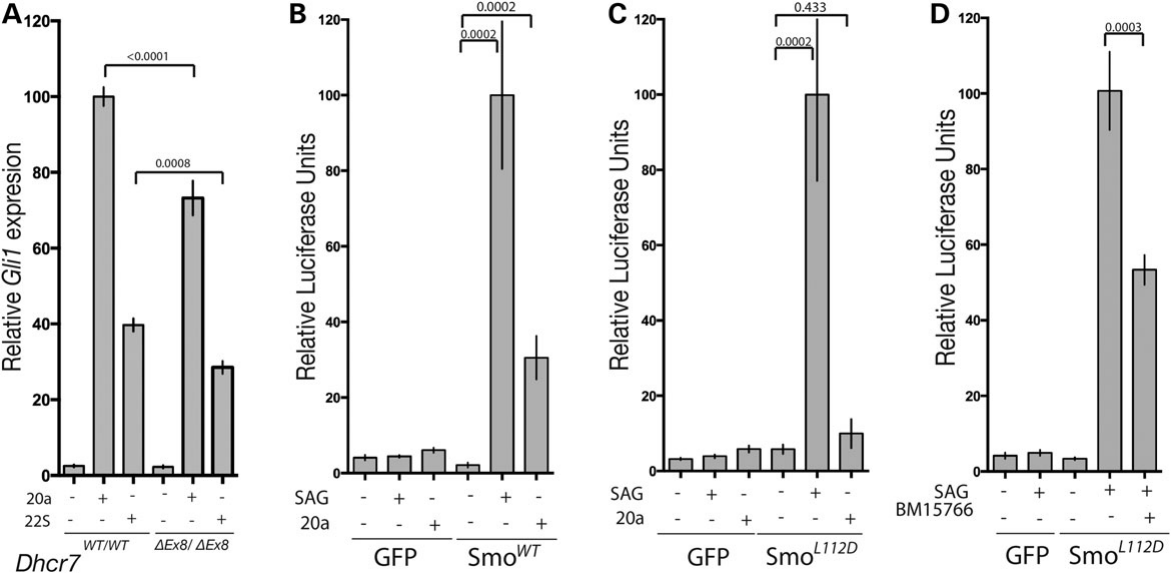
DHCR7 cholesterol biosynthetic activity is required for normal SMO activation by oxysterols. (**A**) *Gli1* transcriptional response to two activating oxysterols was reduced in *Dhcr7^ΔEx8/ΔEx8^* MEFs. Paired *t*-test (*n* = 9). (**B**) The GLI response of NIH3T3 fibroblasts to SAG and 20*α*-hydroxycholesterol depends upon transfection with WT SMO when endogenous SMO is disrupted by CRISPR (*n* = 8). (**C**) Transfection with SmoL112D rescues the GLI response of SMO-CRISPR fibroblasts to SAG, but not 20*α*-hydroxycholesterol (*n* = 8). (**D**) BM15.766 inhibits the GLI-response of SmoL112D transfected SMO-CRISPR fibroblasts (*n* = 15). All panels illustrate mean ± SEM. *P*-values are from Mann–Whitney tests unless stated otherwise.

Specific oxysterols have been shown to modulate the activity and cilia localization of SMO via direct molecular interaction (45,47–49). As cholesterol is itself the precursor for the synthesis of oxysterols, we sought to determine whether the reduced SMO cilia localization observed in the absence of DHCR7 activity could be attributed to reduced oxysterol levels. We first determined whether SHH pathway activation by oxysterols is also sensitive to the absence of DHCR7 enzymatic activity. *Dhcr7^ΔEx8/ΔEx8^* MEFs exhibited reduced SHH pathway response to two oxysterols known to directly activate SMO with varying degrees of potency (44–46) (Fig. 6A), indicating that oxysterols cannot substitute for the reduced cholesterol levels that result in SLOS. To further test whether reduced activation of SMO in the absence of DHCR7 activity occurs independently of oxysterol production, we generated SMO functional-null 3T3 fibroblasts using clustered regularly interspaced short palindromic repeats (CRISPR)-Cas9 and transfected them with SMO expression constructs. SMO-CRISPR fibroblasts regained responsiveness to SAG and oxysterol stimulation when transfected with WT SMO (Fig. 6B). When transfected with an engineered SMO construct that can be activated by SAG, but is unable to be bound and activated by oxysterols (47) (Fig. 6C) the SHH response of SMO-CRISPR cells remained sensitive to DHCR7 inhibition (Fig. 6D). Taken together these data highlight a requirement for the synthesis of cholesterol by DHCR7 for SHH pathway activation that is independent of its function as a precursor for oxysterol ligands of SMO.

## Discussion

There is a substantial body of evidence detailing a range of interactions between SHH signalling and the cholesterol biosynthetic pathway (reviewed in 1,6); however, despite this the molecular pathophysiology of SLOS is unclear. In this study, we demonstrate that the prevalent c.964-1G>C *Dhcr7* mutation perturbs SMO cilia localization and SHH pathway activation as a consequence of reduced cholesterol biosynthesis, and we provide evidence that this occurs independently of reduced oxysterol levels or the accumulation of cholesterol biosynthetic precursors. In addition, we highlight that the widely used DHCR7 inhibitor AY9944 has the potential to inhibit SHH signalling at, or downstream of SMO activation, unlike the structurally unrelated DHCR7 inhibitor BM15.766 which we demonstrate throughout this study to recapitulate the effect of the c.964-1G>C *Dhcr7* mutation.

Our results are consistent with previous studies implicating cholesterol in SHH signalling (48,84). However these studies, which modelled SLOS by depleting cholesterol with cyclodextrin and statins, did not distinguish a requirement for cholesterol and its derivatives from an inhibitory effect of accumulated cholesterol precursors such as 7DHC, as both are depleted by these treatments. As these precursors have previously been proposed to be functionally relevant (55,56), the molecular basis of the SLOS morphological phenotype could not be unambiguously interpreted from these experiments. When we blocked 7DHC production with a statin, we were unable to counteract the negative consequences on SHH signalling resulting from the loss of DHCR7 activity. This suggests that PTC repression of SMO activation is not mediated by 7DHC or its derivative Vitamin D3 as previously proposed (55). Furthermore, PTC-independent activation of SMO via SAG is sensitive to DHCR7 mutation, emphasizing that reduced SHH pathway activity in SLOS is not mediated by PTC. In addition to mediating the SMO inhibitory activity of PTC, accumulation of 7DHC was suspected to contribute to the SLOS phenotype through an ill-defined mechanism (57). As we found no evidence of an inhibitory activity of 7DHC on SHH signal transduction, our study argues against the involvement of 7DHC in causing holoprosencephaly (HPE)-related anomalies, which are the most visible manifestation of SLOS. Consistent with the latter findings, cholesterol rescue of SHH signalling in the absence of DHCR7 activity does not depend on suppression of 7DHC production via feedback inhibition. Additionally, a recent study supports a positive role for 7DHC in SHH signalling that is mediated by 7DHC-derived oxysterols, which like cholesterol-derived oxysterols have a potent stimulatory effect on SMO (48). Taken together, these data demonstrate that defective SHH signalling in SLOS can more readily be explained by a deficit of a metabolic product(s) of DHCR7.

In addition to 7DHC accumulation, transcriptional up-regulation of DHCR7 protein in response to reduced cholesterol levels has also been proposed to inhibit SHH signalling in SLOS (58,59). As transcription of DHCR7 and other enzymes in the cholesterol biosynthetic pathway are regulated by the levels of their precursors and products, depletion of sterols by cyclodextrin and statin treatment has a different effect on their expression when compared with DHCR7 mutation (48,84). In this study, we have overcome these limitations by investigating perturbed SHH signalling in the context of a clinically relevant mutation of *Dhcr7*. This experimental model allowed us to demonstrate that the prevalent c.964-1G>C mutant DHCR7, contrary to previous proposals (58,59), is not a negative regulator of SHH signalling in SLOS. In contrast to the effect of chemical inhibition of DHCR7 activity with AY9944 or BM15766, we observed that c.964-1G>C mutation reduces the level of *Dhcr7* transcript, possibly due to non-sense-mediated decay (82). Furthermore, overexpression of DHCR7 rescued defective SHH signalling rather than attenuating it further.

The role of oxysterols in the regulation of SHH signalling has attracted significant attention (44–48). As cholesterol serves as a precursor for many active oxysterols their levels are predicted to be reduced in the absence of DHCR7 activity, which was thought to explain defective SHH signalling in SLOS. At low concentrations a number of oxysterols act synergistically with SAG to activate SMO via direct molecular interactions at two distinct sites, whereas at higher concentrations oxysterols activate SMO independently of SAG (45,48). We propose that reduced SHH signalling in SLOS is unlikely to be a consequence of reduced oxysterol levels for three reasons. First, synergy is not observed between oxysterols and SHH-induced SMO activation (45). Secondly, perturbed DHCR7 activity affects SMO activation by both SHH and SAG to a comparable extent. Finally, the reduced activation of the pathway by a high concentration of oxysterol in the absence of DHCR7 activity provides a further indication that depleted oxysterol levels cannot explain reduced SHH signalling in SLOS. We, therefore, conclude that the defective SHH response in SLOS occurs independently of oxysterol depletion and results instead from a deficit in the level of cholesterol itself. Cholesterol impacts on SHH signalling at the level of SMO, as SMO cilia localization is diminished in a *Dhcr7* mutant background, whereas a constitutively active SMO mutant is insensitive to inhibition of DHCR7 enzymatic activity. Further support for this hypothesis comes from the observation that an oxysterol-insensitive mutant of SMO remains sensitive to cholesterol depletion due to DHCR7 inhibition. Unlike oxysterols, cholesterol is unable to activate SHH signalling and thus plays a permissive role in the activation of SMO by other ligands, a conclusion supported by another recent study (48). It is currently unknown whether cholesterol regulates SMO activity by directly interacting with it; however, this activity of cholesterol is independent of the SMO oxysterol-binding motif (48). Finally, our study supports the conclusion from studies in rodents and humans that the level of maternal cholesterol transfer during gestation contributes to the severity of symptoms (85–87). Cholesterol delivery *in utero* might, therefore, be an effective intervention to ameliorate SLOS symptoms. Further studies will be necessary to assess this possibility.

## Materials and Methods

### Cell culture

Shh-Light2 cells (a gift from P. Beachy) and NIH3T3 MEFs (obtained from the American Type Culture Collection) were cultured in Dulbecco's modified Eagle's medium (DMEM) containing 4.5 g/l d-glucose and 0.11 g/l sodium pyruvate, supplemented with 2 mm l-glutamine, 100 Units/ml penicillin, 100 µg/ml streptomycin (all Gibco) and 10% new-born calf serum (MP Biomedicals). Primary fibroblasts were derived from individual E13.5 embryos created by mating 129SvEv mice heterozygous for the *Dhcr7^ΔEx8^* allele. Embryos were decapitated and eviscerated, then enzymatically and manually dissociated. Fibroblasts were cultured in DMEM containing 4.5 g/l d-glucose and 0.11 g/l sodium pyruvate, supplemented with 2 mm l-glutamine, 100 Units/ml penicillin, 100 µg/ml streptomycin and 15% fetal calf serum (all Gibco) (referred to as MEF medium). Genotyping of fibroblasts was performed by polymerase chain reaction (PCR). Experiments were performed with fibroblasts isolated from three independent mutant and WT embryos. In all assays described, serum in the culture medium was substituted for ITSX (Gibco) to starve cells of cholesterol and other lipids present in serum and grown to confluence to induce cell-cycle arrest and ciliogenesis.

### Metabolite extraction and sample preparation

Apolar metabolites, including sterols, were extracted from 6 cm plates of confluent fibroblasts as follows. Plates were removed from the culture conditions and immediately transferred to ice cold conditions and culture media removed. Plates were washed twice with ice cold phosphate buffered saline (PBS) and all residual PBS removed. Working quickly to avoid excessive evaporation, cells were scraped into 600 μl water/methanol (1:1) and transferred to a 2 ml Eppendorf tube containing 300 μl chloroform (containing 5 nmol ergosterol as an internal standard). Plates were washed with 600 μl methanol, which was added to the tube. Extracts were vortexed briefly and pulse sonicated (3 × 8 min) in a water-bath sonicator at 4°C for 1 h. Samples were spun (13 200 rpm, 4°C, 10 min), and the supernatant transferred to a new tube before drying in a rotary vacuum concentrator. Apolar and polar metabolites were partitioned by the addition of 700 μl chloroform/methanol/water (1:3:3) and subsequent centrifugation (13 200 rpm, 4°C, 10 min). The lower, apolar phase was transferred to a glass vial, and the remaining polar phase re-extracted with a further 100 μl chloroform. The combined apolar phase was dried, washed twice with methanol and derivatized directly with 20 μl N, O-bis(trimethylsilyl) trifluoroacetamide + trimethylchlorosilane (TMCS) (Thermo Fisher Scientific).

### Gas chromatography-mass spectrometry (GC-MS) and data analysis

Metabolite analysis was performed by GC-MS using an Agilent 7890B-5977A system. Splitless injection (injection temperature 270°C) onto a 30 m + 10 m × 0.25 mm DB-5MS + DG column (Agilent J&W) was used, using helium as the carrier gas, in electron ionization mode. The initial oven temperature was 80°C (2 min), followed by temperature gradients to 140°C at 30°C/min, to 250°C at 5°C/min and then to 320°C at 15°C/min (held for 6 min). Metabolites were identified by comparison with the retention times and mass spectrum of authentic standards using the MassHunter Workstation software (B.06.00 SP01, Agilent Technologies). Abundance was calculated by comparison to responses of known amounts of standards.

### Compounds

Water soluble SAG was purchased from Calbiochem. Shh-N was produced in bacteria according to (88). BM15.766 (Santa Cruz) and AY9944 (Calbiochem**)** were solubilized in Dimethyl sulfoxide (DMSO). BM15.766 was present at a concentration of 15 μm and AY9944 at 10 μm in assays unless stated otherwise. Lovastatin (Sigma) solubilized in H_2_O was present at a concentration of 5 μm in assays. 7DHC, 22(S)-hydroxycholesterol and 20*α*-hydroxycholesterol (all Sigma) were solubilized in ethanol. Cholesterol (Sigma) was solubilized in 30% 2-hydroxypropyl-*β*-cyclodextrin (Sigma). Cholesterol and 7DHC were present at 50 μm, and 22S- and 20*α*-hydroxycholesterol were present at 10 μm in assays. In all cases equivalent concentrations of vehicle were added to controls. When present 2-hydroxypropyl-*β*-cyclodextrin was at a concentration of 0.1%.

### Expression plasmids

The ORF encoding human *Dhcr7* was obtained from Source Bioscience, subcloned into pENTR11 vector (Life Technologies) then recombined into pMXS Gateway (Addgene Plasmid #18656) for retroviral expression. Constitutively active human SmoM2 was a gift from P. Beachy. The L112D substitution was introduced into WT mouse SmoCFP (a gift from J. Jiang) by site-directed mutagenesis. EGFP N2 (Clontech) was used as a transfection control.

### Smoothened CRISPR

Oligonucleotides with sequences CACCGGCGGCGGAGGGCTGGTCAC and AAACGTGACCAGCCCTCCGCCGCC were annealed and cloned into vector px459 (Addgene Plasmid #48139) according to (89) for co-expression of the guide RNA, Cas9 nuclease and puromycin selectable marker. NIH3T3 cells were nucleofected, selected for 48 h with 2 μg/ml puromycin then plated at limiting dilution into 96-well plates. Single cell plating was confirmed and isogenic clones were propagated. The Smo deletion was identified by immunostaining and insensitivity to SAG stimulation was confirmed by luciferase assay.

### Retrovirus infection

Plat-E ecotropic packaging cells (Cell Biolabs) were transfected with pMXS viral expression construct using GeneJuice (Novagen). The following day transfection medium was replaced with the MEF culture medium, which was conditioned with viral particles for 24 h. Of note, 4 μg/ml polybrene was added to viral supernatant diluted 50/50 with the fresh MEF medium and used to infect subconfluent MEFs for 48 h. Transgene expression was confirmed by immunofluorescence using anti-human DHCR7 antibody (Santa Cruz).

### Cilia assays

Fibroblasts were plated on glass coverslips. Following stimulation with Shh-N cells were fixed for 1 h with 4% PFA then permeabilized for 10 min with 0.5% Triton X-100 in PBS, followed by blocking in 1% bovine serum albumin, 0.1% Triton X-100 and overnight incubation with anti-Smo (Abcam) and anti acetylated-tubulin (Sigma). Following secondary antibody staining (Jackson) coverslips were mounted and then imaged with a Leica SP5. Six confocal planes 0.5 μm apart containing ∼200 cells per field were imaged. ImageJ was used for image analysis: the maximum intensity of each pixel across all confocal planes was determined by maximum intensity projection of acquired z-stacks; Acetylated-tubulin staining was used to automatically segment cilia; mean SMO immunostaining per cilia pixel was calculated by dividing the sum of the maximum intensities of each cilium-assigned pixel by the area of the cilium; the value of a manually assigned background pixel was subtracted from all plotted data.

### qPCR assays

Fibroblasts were plated in 48-well plates (Nunc). Cells were stimulated for 16 h in the presence of compounds described in figure legends followed by Trizol extraction of RNA and cDNA synthesis with Superscript3 (Life Technologies). qPCR was performed using an AB 7900HT machine and Platinum SYBR green Supermix. Primers used were: actin F-TGGCTCCTAGCACCATGA, R-CCACCGATCCACACAGAG; Gli1 F-TTATGGAGCAGCCAGAGAGA, R-GAGCCCGCTTCTTTGTTAAT; HMGCR F-GAGCAGCGACATCATCATCC, R-GGCCAGCAATACCCAGAATG; Dhcr7-F-CTGATAGCAGAGGCCCTTTC, R-CCAATCATCGGAGACATCTG; note that the Dhcr7 primer pair used detects the truncated IVS8-1G>C allele. To determine the relative expression level of each gene between samples delta cT values were calculated and normalized to the level of actin.

### Luciferase assays

Shh-Light2 cells were seeded into 96-well plates and cultured until confluent. NIH3T3 cells were seeded into six well plates and transfected using Lipofectamine LTX (Life Technologies) the following day with plasmids encoding the GBS-Luc reporter (90), constitutively expressed TK-Renilla (Promega) for normalization, and expression plasmids indicated in figure legends and detailed below. Transfected cells were then split into 96-well plates. Cells were stimulated for 48 h in the presence of compounds described in figure legends, then lysed and analysed using the Dual Luciferase Reporter Assay (Promega).

### Statistics

All statistical analysis of graphed data was performed using GraphPad Prism 6.

For luciferase assays, triplicate or quadruplicate samples were analysed within each experiment. Three or more experiments were performed and all data points were normalized. Mann–Whitney tests were used to evaluate differences between pooled data collected under each experimental condition.

For qPCR assays, one or two samples were analysed within each experiment and relative expression levels were normalized. Two-tailed paired *t*-tests were used to evaluate differences between the normalized expression values of replicate samples of compared experimental conditions.

For SMO cilia localization assays, two images from each sample were obtained and data presented is combined from three independent experiments. Kolmogorov–Smirnov tests were used to determine *P*-values for the differences between cumulative distributions of pooled data collected under compared experimental conditions.

For GC-MS experiments, triplicate samples were collected and the absolute abundance of cholesterol and 7DHC in each sample was normalized to the average level detected in control fibroblasts. Alternatively, the ratio of absolute levels of 7DHC and cholesterol in each sample was expressed. In cases where multiple independent experiments were performed, data from each experiment was pooled and two-tailed *t*-tests were performed without the assumption of consistent standard deviation (SD).

### Study approval

All procedures performed on mice in this study were carried out according to the United Kingdom Home Office regulations under the project license PPL80/2528 and approved by the Animal Welfare and Ethical Review Panel of the MRC National Institute for Medical Research.

*Conflict of Interest statement*. None declared.

## Funding

This work was supported by a grant from the Simons Foundation (202084, J.J. and J.B.) and by the Medical Research Council (U117560541). J.J. is affiliated to the Oxford Epilepsy Research Group (OxERG). Funding to pay the Open Access publication charges for this article was provided by the Medical Research Council.
